# Enhanced and Prolonged Immunogenicity in Mice of Thermally Stabilized Fatty Acid-Conjugated Vaccine Antigen

**DOI:** 10.3390/vaccines13020168

**Published:** 2025-02-10

**Authors:** Bo Mi Kim, Yeon-Ho Kim, Hai V. Ngo, Hy D. Nguyen, Chulhun Park, Beom-Jin Lee

**Affiliations:** 1 Department of Pharmacy, College of Pharmacy, Ajou University, Suwon 16499, Republic of Korea; kbm001213@ajou.ac.kr (B.M.K.); khc369898@ajou.ac.kr (Y.-H.K.); haingo25895@gmail.com (H.V.N.); hynguyendinh@ajou.ac.kr (H.D.N.); 2 Department of Pharmacy, College of Pharmacy, Jeju National University, Jeju 63243, Republic of Korea; chpark@jejunu.ac.kr; 3 Institute of Pharmaceutical Science and Technology, Ajou University, Suwon 16499, Republic of Korea

**Keywords:** hemagglutinin, vaccine antigen, fattigation platform, fatty acid-conjugated vaccine antigen, thermal stability, prolonged immunogenicity

## Abstract

Background/Objectives: Influenza vaccines require good thermal stability without the need for refrigerator storage. Although the fatty acid-conjugated hemagglutinin (Heg) vaccine antigen provides good stability in both solid and liquid states, its therapeutic effectiveness must be validated in vivo. This study aimed to investigate the immunogenicity of the thermally stabilized Heg-oleic acid conjugate (HOC) and compare it with native Heg as a reference. Method: To evaluate HOC immunogenicity, an enzyme-linked immunosorbent assay was used to measure hemagglutinin inhibition (HI) titers, serum IgG antibody titers (IgG1, IgG2a), and cytokine secretion levels (IFN-γ, IL-4) in BALB/c mice after intramuscular (IM) injection. Results: Thermally stabilized HOC induced higher and more sustained serum IgG1 and IgG2a responses than the native Heg vaccine antigen. IgG1 is typically associated with a Th2 response, whereas IgG2a is associated with a Th1 response. HOC appeared to enhance both responses, inducing a more balanced immune response. Moreover, HOC antigens stimulate broader immune responses, suggesting stronger and longer-lasting immune memory. The cytokine levels of IFN-γ (2.8-fold) and IL-4 (6-fold) were significantly increased in the HOC-immunized group compared to the Heg group. IFN-γ, a cytokine that activates the Th1 immune response, demonstrated the enhanced ability of HOC to induce a Th1 response. IL-4, a cytokine that promotes the Th2 response, indicated that HOC also strongly induced a Th2 response. The thermal stability of HOC antigens was crucial for maintaining their structural integrity, enabling the continuous exposure to the stable antigen without denaturation. This allows immune cells to recognize stable antigens efficiently and form long-term immune memory. Conclusions: The stability of HOC antigens enhanced the antigen processing efficiency of antigen-presenting cells (APCs) and stimulated immune responses. The fatty acid-conjugated vaccine antigen could provide improved storage stability but also enhance immunogenic efficacy compared to the native antigen, supporting its potential for further applications.

## 1. Introduction

Vaccination remains the most effective form of protection against influenza. Neutralizing antibodies are commonly measured to correlate with vaccine-induced protective immunity against influenza [1]. A typical test used to evaluate vaccine response to influenza antigens is hemagglutination inhibition (HI). These standardized tests are easy to perform and quantitatively measure antibodies based on their ability to neutralize viral particles [2]. BALB/c mice typically respond to inactivated influenza and subunit vaccines via a Th2-type immune response associated with stimulation of IgG1 antibodies. However, the predominant antibody isotype presented in the sera of mice that survive viral infections is IgG2a, which is stimulated by a Th1-type immune response. Stimulation with IgG2a antibodies is associated with the increased efficacy of influenza vaccines [3].

Almost all commercially available influenza vaccines contain two surface glycoproteins, hemagglutinin (Heg) and neuraminidase (NA), as the key antigens, with Heg being the dominant antigen [4,5]. Heg has been widely used as a vaccine antigen because it effectively blocks viruses from binding to host cells. However, the immunogenicity of vaccine antigens can be significantly reduced by chemical modifications and unfolding caused by external environmental factors, such as temperature and heat [6].

Because of the issues with the low stability of vaccines at room temperature, influenza vaccines are generally stored under refrigerated conditions (2–8 °C) to prevent potency and efficacy loss. This requirement entails a significant financial burden, as 20–65% of the vaccine price accounts for maintaining the vaccine cold chain; a temperature-controlled supply chain for transporting and storing vaccines to preserve their potency. The cold chain ensures a series of temperature controls during transportation, storage, and distribution of vaccines from the manufacturing site to the final delivery point [7]. However, an established cold chain does not guarantee vaccine quality. Accidental exposure to heat or unintentional freezing of the vaccine during transportation and storage can cause damage. Because of the high cost of maintaining cold chains and managing failures, thermally stable vaccines that do not require refrigeration are highly desirable [8]. Thermally stable vaccines are not only more accessible to populations in developing countries that are lacking cold chain infrastructure, but they also enable their more efficient and widespread distribution in developed countries to combat seasonal and epidemic outbreaks, such as influenza [7,9]. Enhancing vaccine stability to reduce waste and storage costs is therefore of utmost importance [10]. Therefore, influenza vaccines in both solid and liquid states require good thermal stability without storage in refrigerators or cold chambers.

A common approach to thermally stabilize a vaccine is the addition of stabilizing adjuvant [11]. Alumina salt adjuvants are included in vaccines for humans approved by the USFDA (United States Food and Drug Administration, the regulatory agency responsible for the approval and regulation of vaccines and drugs in the US) [12]. However, despite the equipment and practices designed to minimize the risk of the inadvertent freezing of vaccines containing aluminum adjuvants, this risk remains high in both developed and developing countries. This has the potential to have serious consequences, as vaccines damaged by freeze–thaw cycles are likely to be less effective than vaccines that have been properly stored until the time of administration [13]. In addition to stabilizing the adjuvants, vaccines are dried to increase their thermal stability [8]. Lyophilization, a well-established formulation approach in the pharmaceutical industry, is commonly used to stabilize expensive and delicate liquid biological products such as vaccines. However, subsequent dehydration stress during lyophilization often causes stability issues for the proteins and adjuvants in vaccines [14].

Recently, the fattigation platform has emerged as a simple yet innovative technology involving the conjugation of various fatty acids with biomolecules to form amphiphilic structures [15,16]. The chain length of the fatty acids in the fattigation platform significantly influences the physicochemical properties of the conjugated material, which are directly related to thermal stability and physicochemical properties [17]. In addition, the binding of fatty acids to an antigen leads to the formation of an amphiphilic structure, because the hydrophobic chain of the fatty acid conjugates externally to the antigen, ultimately increasing the hydrophobicity of the conjugated antigen [18]. When the conjugated antigen is dispersed in water, proteins undergo self-assembly to minimize the exposure of the hydrophobic chains of fatty acids [19]. During this process, increased hydrophobic interactions enhance the binding affinity between proteins, causing them to become more resistant to unfolding and denaturation at higher temperatures [20]. The thermal stabilization effect of fatty-acid-conjugated Heg (HOC) compared with Heg, in both solid and liquid states, was previously reported in our laboratory [15]. However, additional in vivo data supporting the immunogenicity of the new HOC are required to determine their therapeutic effectiveness.

The aims of this study were to investigate the immunogenicity in BALB/c mice of the thermally stabilized HOC and compare it with native Heg as a reference. To assess the HOC immunogenicity, Heg inhibition (HI) titers, serum IgG antibody titers (IgG1, IgG2a), and cytokine secretion levels (IFN-γ, IL-4) in BALB/c mice after intramuscular (IM) injection were measured.

## 2. Materials and Methods

### 2.1. Materials

Hemagglutinin (Heg; MW = 85 kDa) was purchased from Sino Biological (Beijing, China). Moreover, 1-ethyl-3-(3-dimethylaminopropyl) carbodiimide HCl (EDC. HCl) and sulfo-N-hydroxysuccinimide ester (sulfo-NHS) were obtained from Thermo Scientific (Seoul, Republic of Korea). N, N-Dimethyl formamide (DMF) was obtained from Duksan (Seoul, Republic of Korea). Six-week female BALB/c mice were purchased from SamtaCo (Osan, Republic of Korea). Influenza virus A/Korea/2005/H3N2(KBPV-VR-32) was obtained from the Korea University Virus Bank. Goat anti-mouse IgG1 IgG2 secondary antibody (HRP) was purchased from Invitrogen(#PAI-74421)(#M32207), and chicken red blood cells (RBC) were purchased from Innovative Research. The mouse IFN gamma ELISA Kit was purchased from Abcam(#ab282874), and the Mouse IL-4 ELISA Kit was purchased from Invitrogen(#BMS613).

### 2.2. Synthesis of Hemagglutinin-Oleic Acid Conjugate (HOC)

HOC were prepared according to previously described experimental methods ([15]). Heg (400 µg) was resuspended in 1.6 mL of deionized water (DW), filtered to 0.2 µm. EDC (18.4 mg), and was dissolved in 160 mL of DMF by stirring overnight at 330 rpm to ensure complete dissolution. Oleic acid (30.4 µL) was then added to the 160 mL solution and incubated overnight at 25 °C with a speed of 330 rpm for 30 min. Sulfo-NHS (20.8 mg) was then added, and the reaction was maintained under the same conditions for another 30 min. A total of 200 µL of the activated solution was collected and diluted in two steps for a 100× dilution. Then, 1.6 mL of the diluted solution was added to the Heg vial resuspended in DW. The reaction was carried out for 24 h at 25 °C, with shaking at 330 rpm. The Heg-oleic acid conjugate (HOC) was prepared using 12–14 kDa MWCO dialysis membrane tubing (Sigma-Aldrich Co., St. Louis, MO, USA) in 1 L DW. Purification proceeded at 220 rpm, 25 °C for 24 h with two DW changes. The solutions were then frozen at −70 °C for 6 to 24 h and lyophilized for 24 h to remove organic solvents.

### 2.3. Design and Animal Experiments

#### 2.3.1. Experimental Schedules

**Figure 1:**
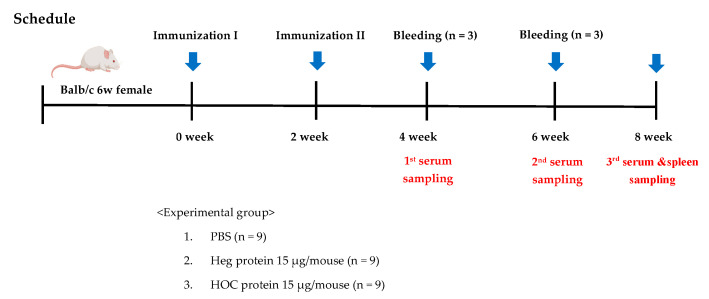
Experimental design and schedule for IM administration of vaccine antigens (Heg or HOC) and samplings in BALB/c mice.

All animal experiments adhered to the Principles of the Care and Use of Laboratory Animals (National Institutes of Health publication no. 85-23, revised in 1985) and received approval (approval number: NPC-240329-V006) from the Institutional Animal Care and Use Committee (IACUC) of NP Chem Bio (Jinju, Kyeongsangnam-do, Republic of Korea). Mice (BALB/c, female, 6 weeks old) were inoculated with Influenza Hemagglutinin (Heg) and thermally stabilized fattigated Heg (HOC) according to an 8-week schedule as shown (Figure 1). Antibody production was then measured.

#### 2.3.2. Establishment of Mice Groups

Three experimental groups were prepared and designed as follows: Group 1, PBS as a negative control; Group 2, native Heg antigen; and Group 3, HOC. On the day of administration, the groups were separated by equalizing the body weights of nine animals in each group.

#### 2.3.3. Injection of Antigen

The first dose was administered on the first day of the study by IM injection of 15 µg for each vaccine antigens (Heg, HOC) in PBS, using a 26-gauge needle in a single 50 µL volume after group separation. The same dose as the first dose was administered again in week 2 of the study. The HOC doses were normalized to those of Heg.

#### 2.3.4. Collection of Blood and Serum

Blood was collected via cardiac aortic sampling from three animals per group at weeks 4, 6, and 8 after administration. After clotting, the blood was centrifuged at 1000× *g* for 10 min, and the supernatant (serum) was collected and stored frozen until the experiments were performed [21].

### 2.4. Hemagglutination and Inhibition Assays for Immune Response Evaluation

#### 2.4.1. Hemagglutination (HA) Assay

Hemagglutinin, a protein essential for the influenza virus to attach and penetrate cells early in the infection, is a glycoprotein present on the surface of the virus. When it encounters red blood cells, it binds to their surface and causes hemagglutination [22]. Influenza viruses exhibit hemagglutination activity, which results in red blood cells sticking together as the virus’s hemagglutinin protein binds to the surface of the red blood cells. When hemagglutination occurred, the plate appeared red and the virus spread across the plate. When hemagglutination was inhibited, the red blood cells settled to form a precipitate in the form of round dots. If enough antigen is present, the red blood cells clump together and spread without separation; however, if the concentration of the antigen is low, the red blood cells settle to the bottom [23]. The ability of the Heg antigen to agglutinate red blood cells was used to determine the antigen titer. A concentration of 8HU was chosen for testing because it exhibits hemagglutination activity without causing hemolysis or inhibition of hemagglutination by the antigen. This was the minimum concentration of the antigen and served as the standard concentration of the virus for use in subsequent experiments.

In a round-shaped 96-well plate, add 50 µL of 0.15 M NaCl to wells (A1 through H9) in rows 2 through 9, except for the first row. Add 100 µL of virus stock solution to one well (A1). Each sample was collected in two wells. Perform 2-fold serial dilutions in columns 2 through 8 and discard the last 50 μL. Use the 9th column as an RBC control. Add 50 µL of the RBC solution to each well, tap the edges of the plate to mix thoroughly, and incubate at room temperature (20–25 °C) for 1 h without shaking [24]. The RBC control was checked to ensure that the RBCs had completely settled and to confirm the results.

#### 2.4.2. Hemagglutination Inhibition (HI) Assay

The hemagglutination inhibition titer was measured using the hemagglutination assay results. Samples used for the HI assay were obtained from three animals per group (PBS, Heg, and HOC) at weeks 4, 6, and 8 after administration. Spleens from each group were isolated and sampled at the end of the experiment at week 8 [25].

In a 96-well plate B through H, add 25 µL of NaCl to each row of wells (B1 through H12). And 50 µL of pretreated 40:1 diluted serum to each well (A1 through A12) in column A. Transfer 25 µL from the first well of each column to the next well in the same row for serial dilution (e.g., A1→B1→C1) and discard the last 25 µL in column H. Add the diluted viral solution to all wells (B1 through H12). Add 25 µL of the diluted virus solution to all wells (A1 through H12); add 25 µL of PBS instead of antigen solution to the serum control plate. Mix with a shaker for 10 s and leave at room temperature (22–25 °C) for 15 min. Add 50 µL of erythrocyte solution to each well, mix, and allow to stand at room temperature for approximately 30 min for complete erythrocyte settling in the control wells. The hemagglutination inhibition (HI) antibody titer was recorded.

### 2.5. Measurement of the Number of Antibodies Specific to the Heg

The ELISA plate was coated with Heg antigen, and the serum sample was reacted with HRP-conjugated IgG1 and IgG2a antibodies, to detect the Heg-specific IgG1 and IgG2a antibodies present in the serum. For the ELISA plate, Heg antigen protein was added to the wells at a concentration of 650 ng/cm^2^ (200 ng/well for 96-well plate) and incubated overnight at 4 °C. The wells were then blocked with 1% BSA for 2 h at room temperature. A total of 100 µL of diluted serum samples (IgG1: 64-fold, IgG2a: 16-fold) were added to Heg-coated plates and reacted for 2 h at 37 °C. The plates were then washed twice with PBS. Plates with serum-bound antibodies were incubated with horseradish peroxidase-conjugated anti-mouse IgG1 (Bethyl Laboratories) or anti-mouse IgG2a (Invitrogen) for 2 h at room temperature and washed at least five times with PBS. At the end of the reaction, the substrate 3,3′,5,5′-Tetramethylbenzidine (TMB) (Invitrogen) was added to the plate, and the reaction was stopped by adding 1 N H_2_SO_4_ when the color reaction occurred. Absorbance was measured at 450 nm using a microplate reader (Molecular Devices) [26]. The IgG1 and IgG2a standards were not measured separately, but the tendency of serum samples to exhibit concentration-dependent absorbance values was confirmed.

### 2.6. Spleen Cell Isolation, Culture, and IFN-γ, IL-4 ELISA Analysis

#### 2.6.1. Spleen Cell Isolation

After blood collection, the spleen was aseptically removed, washed with DMEM, crushed with scissors, filtered through a 100 µm cell strainer, centrifuged (25 °C, 1200 rpm, 5 min), and washed with DMEM. RBC lysis buffer was added to the supernatant to remove red blood cells, reacted at 37 °C for 2 min. PBS was added, and the solution was centrifuged (25 °C, 1200 rpm, 5 min). If red blood cells remained, the above process was repeated, and the final spleen cells were obtained.

#### 2.6.2. Spleen Cell Culture and Antigen Stimulation

Splenocytes were suspended in DMEM containing 20% FBS and 1% antibiotics (penicillin, streptomycin, and gentamicin), and the cells were counted. Splenocytes were seeded in 96-well plates at a density of 5.0 × 106 cells/mL in a volume of 100 µL and treated with 50 µL of Heg antigen to a final concentration of 10 µg/mL (50 µL of antigen at a concentration of 40 µg/mL was treated). Treatments were performed in duplicates for each group. After treatment, 50 µL of culture medium was added, bringing the volume to 200 µL. For the IFN-γ measurements, the cells were incubated in a 37 °C, 5% CO_2_ incubator for 144 h. For the IL-4 measurements, cells were incubated for 24 h at 37 °C, in a 5% CO_2_ incubator [27]. After incubation, the cultures were harvested, and the supernatant was collected by centrifugation and used for the IFN-γ and IL-4 ELISA assays.

#### 2.6.3. IFN-γ and IL-4 ELISA Assays After Splenocyte Antigen Stimulation

IFN-γ (ab282874) and IL-4 (BMS613) ELISA assays were performed with cultures obtained after splenocyte antigen stimulation. IFN-γ and IL-4 ELISA kits were commercially available. The assays were conducted according to the provided protocols summarized below. The IFN-γ and IL-4 standards provided in the kit were prepared by serial dilution. The prepared splenocyte culture aliquots were appropriately diluted and added to the microplate strips, followed by incubation with an antibody cocktail. After sealing, the plates were shaken and incubated at 25 °C for 40 min. Washing buffer was added to each well and the aspiration process was repeated three times. The TMB development solution was added to each well and incubated for 5–20 min at room temperature in the dark. A stop solution was added to each well and shaken for approximately 1 min, and the absorbance was measured at 450 nm.

### 2.7. Statistics

Statistical analysis was performed using analysis of variance (ANOVA) with SigmaPlot software (version 12.5). Statistical significance between samples was considered when the *p* value of the difference did not exceed 0.05 (*p* < 0.001, ***; *p* < 0.01, **; *p* < 0.05, *; ns, no significant difference). All results are expressed as mean ± standard deviation (SD).

## 3. Results

### 3.1. Hemagglutination Inhibitor Antibody (HI Titer)

**Figure 2:**
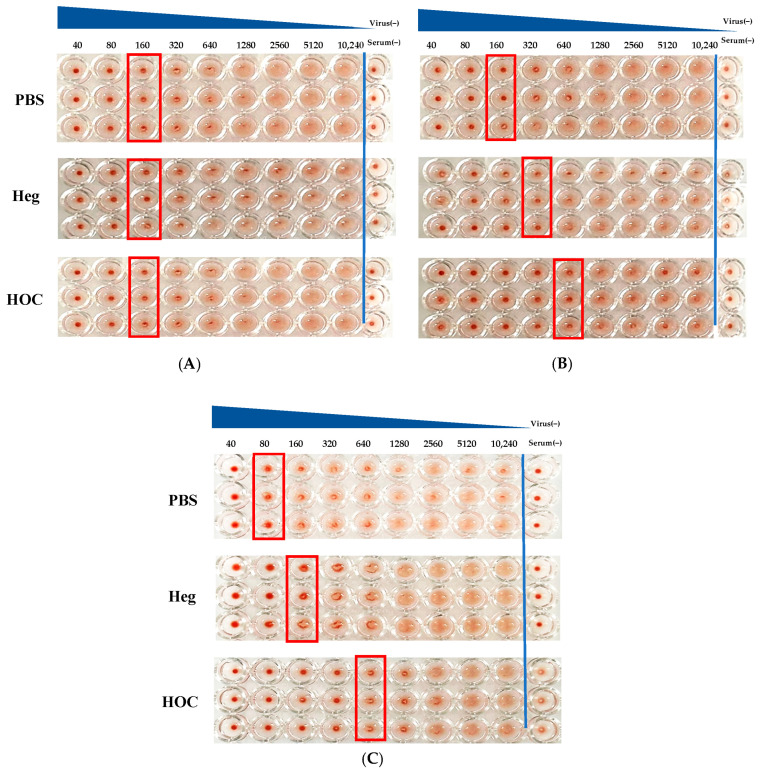
The visual changes in hemagglutination inhibitor (HI) antibody titers in mouse serum samples (×40 dilution) after IM administration of vaccine antigens (Heg, and HOC) during the 1st (**A**), 2nd (**B**), and 3rd (**C**) serum sampling periods (n = 3). The red boxes indicate the antibody titer, representing the highest dilution where hemagglutination is observed. The blue line separates the virus-positive wells from the serum-negative control wells to distinguish the results more clearly.

Serum antibodies inhibit red blood cell aggregation when they bind to Heg cells of the influenza virus. Serum HI titers, which measure the magnitude of the antibody response, are the primary correlates of protection currently used to assess the efficacy of seasonal and pre-pandemic influenza vaccines in humans. The HI test determines antibody titers by identifying the lowest serum dilution required to prevent red blood cell aggregation as viral concentration is a key variable that affects the assay outcome [28]. A higher titer value compared to the control indicates greater efficacy in hemagglutination inhibition. The evaluation of hemagglutination inhibition titers, using a minimum 40-fold dilution of serum, showed that the PBS, Heg, and HOC immune groups had an identical HI titer of 160 at 1st (Figure 2A). The serum results from second sampling period (Figure 2B) showed that the PBS group still showed no agglutination inhibition, whereas the Heg group showed agglutination inhibition up to approximately 320 HI, indicating an enhanced immune response. In the HOC group, agglutination inhibition was confirmed to be 640 HI, and antibody titers were higher in HOC than in Heg cells, indicating a steadily increasing immune response. Serum from the third sampling period showed hemagglutination inhibition titers of 80 HI in the PBS group, 160 HI in the Heg-immunized group, and 640 HI in the HOC-immunized group (Figure 2C).

The hemagglutination inhibition titers in the PBS group were 160 and 80 HI in the second and third serum samples, respectively, which were lower than those in the Heg and HOC immune groups. In other words, the PBS control group exhibited a weak immune response. The Heg immune group showed a titer of 320 HI after the second dose and 160 HI after the third serum dose, which was consistent with the ELISA results described above, indicating that the titer decreased with the duration of administration. In contrast, in the HOC immune group, the antibody titer in the serum remained at 640 HI after the second and third blood draws, indicating a significantly higher HI titer in the HOC immune group than in the Heg immune group, in addition to the maintenance of the titer. This confirms that the HOC antigen induced a stronger immune response than the Heg antigen. Additionally, antibody titers against HOC increased over time, reaching their highest levels at the third blood draw, which is associated with a memory response by the immune system.

Upon exposure to an antigen for the first time after vaccination, the immune system recognizes the antigen and initiates a slow immune response [29]. The initial slow response results in relatively low antibody levels. Upon re-exposure, memory B cells are rapidly activated, producing large amounts of high-affinity antibodies [30]. Antibody titers increased from the first to the third sampling period, likely due to the activation of memory cells formed during the initial immune response. In particular, HOC induced higher levels of antibody production and affinity, suggesting that it created a strong immune memory.

The strong immune response induced by the HOC antigen can be attributed to the thermal stability of the antigens, which contributed to the efficient stimulation of the immune system. Thermal stability is important for maintaining the structural integrity of antigens and ensuring that they are consistently present in the immune system. The stable structure of the HOC antigen likely facilitates an improved interaction with immune cells, particularly B cells, leading to robust immune response [31]. This is supported by the fact that antibody titers to HOC antigens increase over time, indicating the activation of memory B cells and stronger immune memory. These results suggest that the HOC antigen not only induces an immediate immune response but also establishes a lasting immune memory, ensuring long-term protection.

**Figure 3:**
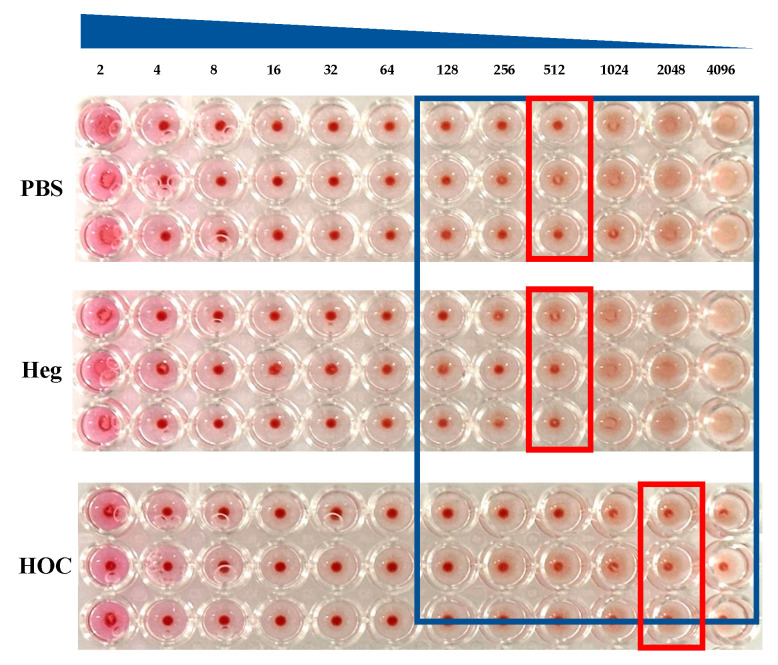
The visual changes in hemagglutination inhibitor (HI) antibody titers in spleen homogenate after the 3rd sampling period, following an IM administration of vaccine antigens (Heg and HOC) (n = 3). The red boxes indicate the antibody titer, representing the highest dilution where hemagglutination is observed. The blue box highlights a specific dilution range to compare antibody titers among PBS, Heg, and HOC groups, emphasizing differences in hemagglutination patterns.

HI antibody titers were measured in spleen homogenates at the third sampling period. The spleen is an immunologically important lymphoid organ that plays key roles in antibody production and memory cell activation. In the spleen homogenate, the aggregation inhibition titer was 512 HI in both the PBS group and the Heg-immunized group, whereas it was 2048 HI in the HOC-immunized group, indicating a significantly higher hemagglutination inhibition titer in the HOC-immunized group than that in the Heg-immunized group (Figure 3).

As the antigen reaches the spleen, B cells recognize it through specific receptors, internalize it, and process it. Upon antigen presentation, B cells interact with helper T cells and are activated. Activated B cells differentiate into plasma cells, which produce different types of antibodies such as IgM and IgG. During this process, B cells increase their specific antibody titers to the antigen and eventually differentiate into memory B cells, which form a long-term immune memory [32]. HOC antigens are more effective for antigen presentation and immune cell activation in the spleen because of their thermal stability. Heat-stable HOC antigens facilitate antigen processing in the spleen and do not alter their structure, thereby providing continuous stimulation to the immune system. This prolonged interaction of HOC antigens with immune cells promotes the continuous activation of memory B cells and plasma cells, suggesting that HOC antigens activate more memory B cells in the spleen and induce them to produce more antibodies. The thermal stability of HOC antigens enables the better exposure of the immune system, increasing the strength and persistence of the immune response. As a result, HOC antigens induced stronger B cell differentiation, increased antibody production, and contributed to the formation of strong immune memory in the spleen.

### 3.2. Heg Protein-Specific IgG Titers by ELISA

**Figure 4:**
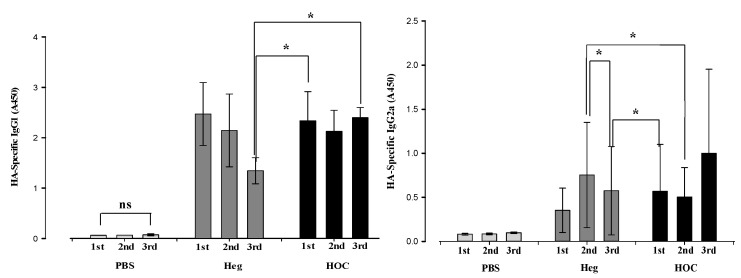
HA-specific IgG1 and IgG2a levels via ELISA assay in mouse serum samples after IM administration of vaccine antigens (Heg, HOC) (n = 3). * *p* < 0.05; ns: no significant difference.

Table 1 and Figure 4 present the results of antigen-specific IgG1 and IgG2a expression in blood samples. IgG1 is predominantly associated with humoral immune (Th2) responses, while IgG2a is linked to cellular immune (Th1) responses [33].

In the PBS group, the absorbance of Heg antigen-specific IgG1 and IgG2a remained low and unchanged over the three sampling times. This indicated that PBS treatment had little effect on antibody production. In the Heg group, IgG1 absorbance increased in the first sampling period but gradually decreased by the third. In contrast, the HOC-immunized group maintained high IgG1 absorbance in the third sampling period. Heg antigen-specific IgG2a expression was absent in the sera of the PBS group in the first, second, and third sampling periods, whereas significant increase was observed at all third sampling periods for the Heg- and HOC-immunized groups. Similarly to IgG1, the mean expression of IgG2a decreased in the third sampling period for the Heg-immunized group, whereas it was higher in the third sampling period for the HOC-immunized group. As a result, Heg stimulates both IgG1 and IgG2a in the early immune response (1st–2nd) but decreases over time. HOC stimulates a sustained immune response in IgG2a with a stable induction of IgG1, showing that HOC can induce a more balanced immune response (Th1/Th2).

### 3.3. Spleen IFN-γ, IL-4 ELISA Analysis

#### IFN-γ and IL-4 ELISA Assay After Splenocyte Antigen Stimulation

**Figure 5:**
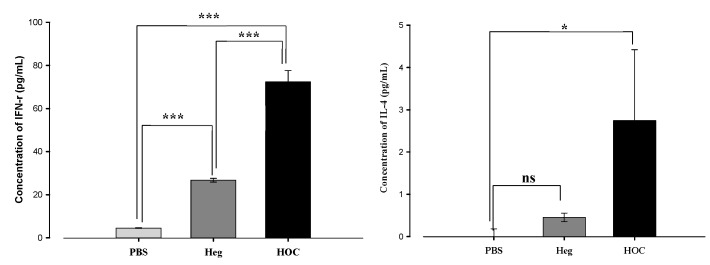
The concentration (pg/mL) of IFN-γ and IL-4 expression in splenocytes isolated from sacrificed mice during the 3rd sampling periods (n = 3). *** *p* < 0.001; * *p* < 0.05; ns: no significant difference.

Cytokines such as IFN-γ and IL-4 mediate immunoglobulin class switching in B cells to IgG1 and IgG2a, respectively, serving as indirect indicators of T helper subpopulations, which are enhanced by changes in the Th1/Th2 balance [34]. Table 2 and Figure 5 show the levels of secretion of IL-4 and IFN-γ in splenocytes. IL-4 is a key cytokine in the Th2 immune response that contributes to humoral immunity and antibody production. IFN-γ is the main cytokine of the Th1 immune response and is associated with macrophage activation and the enhanced function of cytotoxic T cells in cellular immunity [35]

The IL-4 expression in splenocytes was assessed, and no IL-4 expression was observed in the PBS group, whereas increased expression of IL-4 was observed in the Heg-immunized group. In the HOC-immunized group, a 6-fold increase in IL-4 expression was observed compared with that in the Heg-immunized group. When INF-γ expression was checked in splenocytes, very weak IFN-γ expression was observed in the PBS group. A 2.7-fold increase in INF-γ expression was observed in the HOC group compared to Heg.

The increased IL-4 in HOC compared to that in PBS indicated that HOC effectively induced a Th2 immune response. Looking at the IFN- γ values, Heg enhanced the Th1 response compared to PBS, but not as strongly as HOC. HOC induced an approximately 16-fold increase compared to PBS and an approximately 3-fold increase compared to Heg. This indicates that HOC strongly activates the Th1 immune response. Heg primarily activated the Th1 response but has minimal effect on the Th2 response. In contrast, HOC activates both Th1 and Th2 immunity, with particularly strong induction of the Th1 response (IFN-γ). This suggests that HOC may have a balanced immunomodulatory effect, with favorable properties for enhancing cellular immunity.

## 4. Discussion

The HI assay is simple, sensitive, inexpensive, and rapid; therefore, it is often the method of choice for assaying the presence, function, and concentration of antibodies in serum to block the HA protein, thereby inhibiting hemagglutination and providing an indication of the amount of protection against the virus in serum [23,36]. The PBS group was set as a negative control group with little immune response, while the Heg group was selected as a positive vaccine antigen. The difference in antibody titers between PBS group and Heg group was statistically significant (*p* < 0.05), suggesting that Heg significantly induced much higher antibody titers compared to PBS. Furthermore, it was evident that the HOC immunization group produced significantly higher HI titers than the PBS and Heg groups, inducing a stronger humoral immune response. When comparing the amount of IgG antibodies, the amount of Heg antibodies tended to decrease over time, whereas HOC antibodies tended to increase, indicating that HOC induces a stronger and more sustained immune response. The immunogenicity of specific IgG subclasses in the serum showed that HOC maintained a relatively high Th1/Th2 immune response. This balance is critical in ensuring effective immunity, as Th1-mediated responses provide cell-mediated immunity, while Th2-mediated responses support humoral immunity. These results suggested that HOC has some potential as a promising vaccine candidate by enhancing both humoral and cellular immune responses. The sustained production of IgG antibodies and the balanced Th1/Th2 immune response observed in the HOC group could indicate the potential for long-term and robust protection against influenza virus infections.

The concentration of cytokines (IFNγ, IL-4) in splenocytes was also higher in HOC than in PBS or Heg, demonstrating that HOC effectively activated both cellular and humoral immune pathways. It was reported that the splenic lymphocyte proliferation was also measured using HI antibody titration, facilitating greater upregulation after the intranasal vaccination of the inoculated virus by irradiation [25]. While serum measures systemically circulating antibodies, spleen homogenate can assess the function of antibodies locally produced in the spleen. For this reason, the effect of the immune response in the spleen was evaluated by confirming whether the Heg or HOC antigen-specific antibodies are actually effective in inhibiting virus-erythrocyte agglutination after IM injection. The comparison of HI titers of serum and spleen could provide an insight into the systemic and local immune responses of Heg and HOC. This can be meaningful in directly analyzing the presence and function of antibodies in the spleen. Although there has been no previous precedent for performing the HI assay in spleen homogenate, it suggests that measuring HI titers in spleen homogenate and analyzing cytokines with spleen cells can be used to evaluate the immune response induced by vaccines in various ways. The elevated cytokine levels in splenocytes further emphasize the comprehensive immune activation achieved by HOC, which is crucial for effective vaccine-induced protection. However, further investigational studies will be required to validate the effectiveness and reproducibility of this method.

For the first study, the HOC vaccine antigen was found to provide sustained IgG production and balanced Th1/Th2 immune responses. The increased levels of cytokines (IFNγ, IL-4) measured in the spleen homogenate also provide evidence for the effective immunogenic activation of HOC. For these reasons, the HOC vaccine antigen can be used as a potent immunogenic and long-term protection against influenza infection. However, further investigational studies on HOC, utilizing lethal or inoculated influenza viruses, will be carried out to assess its actual protective immunogenic efficacy.

## 5. Conclusions

The current HOC provides important implications and potentials for solving the need for thermally stabilized vaccines that are resistant to temperature changes during distribution and storage. Most of all, thermally stabilized HOC has the potential to not only induce efficient antibody responses but also provide a prolonged immune response. The potential immunogenic capability of HOC as a promising vaccine candidate was highlighted in this study, confirming its enhanced thermal stability and enhanced and prolonged humoral and cellular immune responses. In addition, the persistence of the immune response induced by HOC can be a great advantage in future vaccine development and immunogenic therapy. Finally, the current fattigation platform can be applied for vaccine delivery to increase thermal stability, sustain the immunogenicity, and develop new therapeutically efficient vaccines with diverse antigens.

## Figures and Tables

**Table 1:** The measurement of absorbance for the expression of IgG1 and IgG2a in mouse serum at three sampling periods (n = 3).

Absorbance (450 nm)	PBS			Heg			HOC		
	1st	2nd	3rd	1st	2nd	3rd	1st	2nd	3rd
IgG1	0.06 ± 0.0	0.07 ± 0.0	0.07 ± 0.02	2.47 ± 0.62	2.15 ± 0.72	1.34 ± 0.26	2.34 ± 0.58	2.13 ± 0.42	2.40 ± 0.20
IgG2a	0.08 ± 0.01	0.09 ± 0.01	0.10 ± 0.01	0.35 ± 0.25	0.75 ± 0.60	0.57 ± 0.50	0.57 ± 0.53	0.50 ± 0.33	1.00 ± 0.95

**Table 2:** The measurement of absorbance for the expression levels (pg/mL) of IL-4 and IFN-γ in mouse splenocytes (n = 3).

Cytokines	PBS	Heg	HOC
**IFN-γ**	4.53 ± 0.16	26.74 ± 0.88	72.40 ± 5.27
**IL-4**	0.071 ± 0.018	0.45 ± 0.10	2.74 ± 1.68

## Data Availability

Data are contained within the article.
